# Social Determinants of Health and Happiness of Older Adults in Ghana: Secondary Analysis of Ghana SAGE Wave 2 Longitudinal Data

**DOI:** 10.21203/rs.3.rs-3224059/v1

**Published:** 2023-08-08

**Authors:** Joseph Kojo Oduro, Mary Ama Oduro, Edward Kwabena Ameyaw

**Affiliations:** University of Cape Coast; University of Cape Coast; Institute of Policy Studies and School of Graduate Studies, Lingnan University, Tuen Mun, Hong Kong

**Keywords:** Older Adults, Wellbeing, Happiness, Social Determinants of Health, Ghana

## Abstract

**Background:**

Social determinants of health [SDOH] and happiness have received meaningful consideration as foundational concepts in the field of public health. However, the relationship between the SDOH and happiness of older adults have not received the requisite recognition in Ghana. This study examined the relationship between the SDOH and happiness of older adults in Ghana.

**Methods:**

The study used data from the 2014/2015 Ghana Study on Global Ageing and Adult Health (SAGE) Wave 2. Data was analysed using the Structural Equation Modeling (SEM) technique to investigate the direct, indirect and covariances of the SDOH and happiness of older adults.

**Results:**

The results showed positive relationship between the SDOH and happiness among older adults. The economic stability (*β* = 0.07), neighbourhood and built environment (*β* = 0.02, *P* < 0.001), access to quality education (*β* = 0.56, *P* < 0.01), access to healthy food (*β* = 0.48, *P* < 0.001) social and community context (*β* = 0.41, *P* < 0.05), and access to quality healthcare (*β* = 0.80, *P* < 0.001) had direct relationship with happiness of the older adults in Ghana.

**Conclusion:**

This study shows that the conditions in which older adults were born, live, learn, work, play, worship, and age (SDOH) positively impact their happiness in later life. Neighbourhood and physical environment influence the effect of quality education on happiness of older adults. Social policies and interventions aiming at happiness of older adults should consider the social determinants of health and the mediating effects of food on happiness through quality education, and quality of healthcare system.

## Introduction

Social determinants of health (SDOH) and happiness of older adults is a pivotal area in healthy ageing. The past decades have received noteworthy consideration of the concept of happiness in the field of public health [1]. However, the relationship between the SDOH and happiness concerning older adults is yet to be accorded adequate attention. Therefore, an evidential understanding of the correlation between these two vital concepts as far as older adults are concerned is appropriate and timely. This study drove on the premise that environments in which older adults are born, live, learn, work, play, worship, and age affect a wide range of health, functioning, and quality-of-life outcomes as well as happiness of older adults [2, 3]. In the conceptualization of Health People [2], SDOH comprised five indicators including economic stability, education access and quality, healthcare access and quality, neighbourhood and built environment, and social and community context [2]. It is worth noting that these indicators of SDOH have enormous consequences on livelihoods linked with material affluence, physical safety and subjective life satisfaction which may affect one’s level of happiness [4–7], in this case older adults.

Earlier studies have shown the relationships between the specific indicators of SDOH and happiness [8–11]. There is positive relationship between economic stability and happiness [10, 11]. Many people struggle to have or keep a job but those with the opportunity to have a steady employment are very less likely to live in poverty and very likely to be healthy and happy. Also, working full or part time and earning higher income help people including vulnerable groups like older adults to pay for food, housing, healthcare etc. This enhances happiness among older adults in low- and middle-income countries [10–12] indicating that older adults who are working and receive better income are happier than their counterparts who do not have the opportunity of working to earn for basic living [13, 1, 14].

Besides, access to high quality healthcare services contributes positively towards healthy living and happiness [15]. Thus, availability and access to the needed healthcare services and the satisfaction with health care delivery timely and of high quality are regarded to improve health and brings happiness [16–19]. The implications are that access to healthcare without satisfaction of the care services delivered negatively affect patients’ happiness. For instance, health workers’ intermittent late reporting to work, conclusive diagnosis without listening to patients’ concerns, improper examination of patients, not explaining the diagnosis, shortage of medicines, and medical equipment are negatively associated with happiness of patients [20]. Also, older adults without health insurance could have less access to primary care provider which could also limit their ability to pay for healthcare services and the medications they need. This could negatively impact their wellbeing and happiness.

Furthermore, the neighbourhood and built environment [NPE] or living in a healthy and safer location is linked to health and wellbeing [21 –24]. Living in a neighbourhood that has high rates of violence, unsafe water and air alongside other social health and safety risks has adverse effect on healthy living and happiness of older adults [25]. On the other hand, healthy and happy people are found in a safer environment where there is good interactions among the people [23]. Also, the place of residence is reported to play a significant role in a happy life [21]. Living in a rural or urban area impacts happiness [21]. Access to safe, open, natural, and green spaces, which are designed features for allowing social interaction is associated with happiness [24].

Evidence shows that educational attainment increases the level of happiness through independence and freedom of choice [26]. Highly educated people build better self-esteem and have confidence when compared with those with lower level of educational attainment, which result in higher levels of happiness [27, 28]. Also, higher educational achievement is accompanied by decent occupation, and higher income which impact individual happiness [29, 30].

Imperatively, close relationships and interactions with family, friends, colleagues at work, and community members have key impact on people’s happiness. Access to safer open social spaces for recreational purposes like the community centers, aged friendly drinking spots, church places, and places where older adults can gather to play games are very important characteristics of the social and community context that are directly related to happiness [31]. These enhance interpersonal relationships among older adults [31] and the sense of community which significantly influence happiness [32]. Older adults who have positive relationships at home, at work, and in the community have less to worry about in life. Further, spending time with friends and good neighbours, community participation, and trust in people in the neighbourhood including the local police have significant relationship with happiness of older adults [33]. Also, religiosity and attending religious activities (social integration and engagement) have positive relationship with happiness [34, 35]. An indication that the community serves as social environment that has profound effects on the kind of social support one could get [36, 37]. Aged people are happier when they are socially integrated and engaged into the community where they live[38].

Studies in Ghana have shown that family social capital, including family sense of belonging, autonomy support, control, and social support, vary with self-reported happiness [39]. Happiness among Ghanaians is influenced by several factors such as economic, cultural, social capital and health factors [40]. A wide range of scholarly work have contributed to the debate on SDOH and happiness. Yet these studies do not primarily target older adults. Also, the categorization of the SDOH by the Healthy People [2] as applied in this study does not include access to healthy food or diet which has been shown to have influence on happiness. Lastly, none of the available studies have investigated the direct, indirect and covariance effect of the indicators of SDOH on happiness of older adults in Ghana, as far as our search revealed. Premised on the foregoing, this study examined the relationship between the SDOH and happiness of older adults in Ghana. This is because the conditions in which they are born, live, learn, work, play, worship, and age could affect their happiness as previous studies have shown from different contexts.

## Theoretical perspective

**Figure 1:**
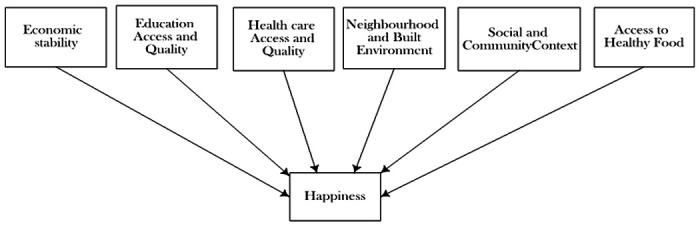
Theoretical model of Social Determinants of Health and Happiness

The Objective List Theory (OLT) strengthened the premise of this study. The theory holds that happiness consists of a human life that achieves certain things from a list of worthwhile pursuits which may include career accomplishments, friendship, freedom from disease and pain, material comforts, civic spirit, beauty, education, love, knowledge, and good conscience [41, 42]. The OLT best explains relationship between happiness and the SDOH as the main aim of this study. Aspects that have applied the OLT include the argument that people’s objectivist judgments would recognise knowledge as an element of happiness because it involves appropriately justified beliefs about meaningful truths [43]. Parker [44] asserts that the most worthwhile lives are those high in various objective goods which principally include happiness and meaning. Although these studies somewhat justify the tenets of the OLT, they apply the theory either making detail explanations of the theory itself or scarcely apply it to older adults in terms of their happiness. Therefore, the relationships between happiness and the SDOH concerning older adults is left of out. The study theorised that happiness of older adults is influenced by the SDoH as categorized by Healthy People [2]. In addition to the five indicators by Healthy People, access to healthy food was included and tested in this study. Figure 1 presents details of the theoretical framework.

## Materials and Methods

### Data source

The data used for this study was obtained from the 2014 Ghana Study on Global Ageing and Adult Health (SAGE) Wave 2, which is supported by the US National Institute on Ageing, Division of Behavioural and Social Research and National Governments. The core SAGE collects a comprehensive data on adults aged 18+, with an emphasis on populations aged 50+, from nationally representative samples in six countries: China, Ghana, India, Mexico, Russian Federation and South Africa. The SAGE Wave 2 for Ghana focused on the health and wellbeing as well as happiness level of older adults (50+) adults. SAGE surveys follow the standard procedures (i.e., sampling, questionnaire development, data collection, cleaning, coding and analysis) which allow cross-country comparison. The survey employs a stratified two-stage sampling technique. The initial stage involves the selection of Primary Sampling Units (PSUs) by region and location (urban/rural) across the six countries. The second stage involves the systematic selection of enumeration areas (EAs), households (HH) with 50 + adults, and households (HH) with persons aged 18–49. For this study, only those 50 years and over (n = 781) who had complete information on the variables of interest were included [13]. Respondents were classified into four categories of functional age brackets: the “younger old” (50–64 years) “young old” (65–74 years); the “old-old” (75–84 years); and the “oldest old” (85 years and above) [45]. The data was deemed suitable for this study because it is nationally representative.

## Measures

### Dependent variable

#### Happiness

In general, the dependent variable assessed if respondents lived happy life or were satisfied with life. This was measured by three variables including satisfaction with living condition (1 = very satisfied to 5 = very dissatisfied), overall quality of life (from 1 = very good to 5 = very bad), and level of happiness (from 1 = very happy to 5 = very unhappy). For the analysis of this study, “happy in life” was recoded into a binary variable, “very happy”, “happy”, and “moderately happy” were coded into 1 representing older adults who rated themselves as happy (1 = happy) while “unhappy” and “very unhappy” coded into 0 indication older adults who rated themselves as unhappy (0 = unhappy).

### Independent variables

#### Social determinants of health:

The independent variables as adopted from the Healthy People [2] were divided into five categories including: (a) economic stability, (b) access to quality education, (c) access to quality healthcare, (d) neighbourhood and built environment, as well as (e) social and community context. Some of the variables measuring these indicators were renamed/recoded to reflect items in the SDOH model and to suit the analysis.

*Economic stability* denotes living out of poverty to afford basic things like healthy food, healthcare, housing, quality education. It was defined in this study as having steady employment and earning enough to pay for food, housing, health care, and education can reduce poverty and improve health and well-being. It was measured in this study by earnings/income (yes/no), currently working, renamed employment (yes/no).

*Access to quality education* is where learners who are well-nourished, are ready to participate and learn, are supported by their families and communities; healthy, safe, and supportive environments. For the analysis, it was defined as high-quality educational achievement and or opportunities for older adults in their entire life environment. it was measured by highest level of education (from 1 = Primary/JHS to 3 = Tertiary), and years educated (from 1 = < 10 years to 4 = > 30 years).

*Access to quality healthcare* is an indication that an individual gets the healthcare services needed where they live. In this study, it was considered as older adults’ access to timely and high-quality healthcare services in the places where they have spent their entire lives. It was measured to include healthcare provider (from 1 = medical doctor to 7 = home health care worker), quality of healthcare (from 1 = very good to 5 = very bad), and satisfaction with quality of care (from 1 = very satisfied to 5 = very dissatisfied).

*Neighbourhood and built environment* (NBE) are the areas that promote the health and wellbeing of and individual. For this study, NBE denote improved health and safety in the place where older adults are born, grow, live, learn, work, play, worship, and aged. it comprised place of residence (rural/urban), always lived here in the community, renamed childhood residence (yes/no), as well as safe on the street, renamed safety (from 1 = completely safe to 5 = not safe).

*Social and community context* **(SCC)** refer to the relationships and interactions with family, friends, working colleagues and community members. In this study it is defined as the social support that older adults need in the places where they were born, live, learn, work, play, worship, and aged/grow up. It was measured by community meetings renamed social integration (from 1 = never to 5 = daily), religious services renamed community engagement (from 1 = never to 5 = daily) and feel left out which was renamed social isolation (from 1 = never to 4 = often).

*Access to healthy food* included number of fruit servings per a day, and number of servings of vegetable per a day, renamed fruit and vegetable consumption (from 1 = < 2 servings to 4 = > 6 servings), hungry, no money to buy food (from 1 = every month to 5 = never).

### Confounders

Three confounders were included in this study. These were sex (1 = male to 2 = female), age (1 = 50–64[younger old, 2 = 65–74[young old], 3 = 75–84 [old old, 4 = 85+ [oldest old]), and marital status (1 = Never married, 2 = Married, 3 = Separated/divorced/cohabiting, 4 = Widowed).

### Data analysis

**Figure 2:**
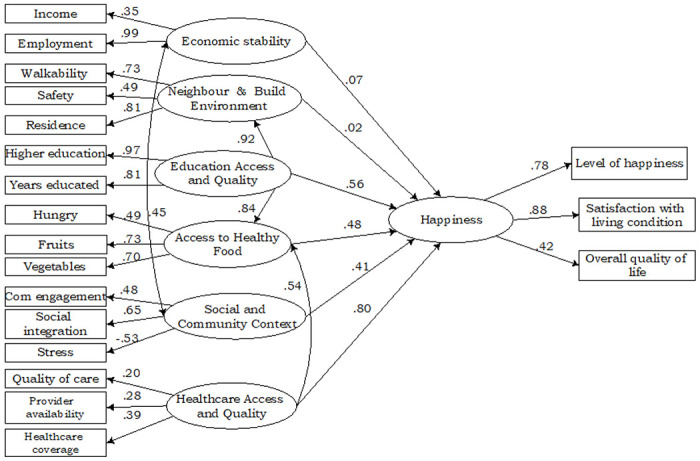
Structural Equation Modelling of Social Determinants of Health and Happiness

Statistical analyses were performed with SPSS version 26 and Amos version 23. Data analysis was conducted by the used of the cross-tabulation, Confirmatory Factor Analysis (CFA) and the Structural Equation Modelling (SEM) techniques. First, the chi-square test was used to examine if the percentage distribution of the older adults who were happy in life were statistically significantly (p < 0.05) different by the selected background characteristics. SEM uses factor analysis to create an indicator score to measure a latent variable. Therefore, SEM was applied because the indicators used to measure the SDOH, and happiness were latent. The model was setup using a theory-based conceptual model (see Fig. 1) used for the study. At the CFA level, the model fit for the latent variables was assessed, and were grouped into five: economic stability, education access and quality, healthcare access and quality, neighbourhood and built environment, and social and community context according to the theory-based conceptual model for the analysis in this study. The CFA was therefore used to determine the measured variables that shared common variance and defined a theoretically sound construct or latent variable. Measures that loaded unto one factor and effectively explained the variance were retained (See Fig. 2). Also, the CFA was used to determine the statistically significant levels of the variables. The models were fitted using the maximum likelihood estimation approach. At the final stage of the model, modification indices were computed to improve the fit of each latent variable. The goodness-of-fit of the model was evaluated using the chi-square (χ^2^) test, the Non-normed Fit Index (NFI), Tucker-Lewis Index (TLI), Comparative Fit Index (CFI), and the Root Mean Square Error of Approximation (RMSEA). All the three relative fit indices (NFI, TLI, and CFI) exceeded the 0,90 criterion [46]. The value of RMSEA was also lower than 0.08 [46]. All the variables measuring the latent variables were significant at 1% level of significance (*P*< 0.01) in the modified hypothesised model.

## Results

Approximately 56 percent of older adults were females with 63.3 percent being relatively youngest (50–64 years). Sixty percent were married, 50.6 percent had attained Secondary/SHS education, 49.9 and 56.3 percent had 2 to 3 servings of fruits and vegetables respectively. Majority of older adults (76.8%) had lived in the same community/neighbourhood from childhood. Seventy one percent were working, and 68 percent were earning income. Almost all older adults (91.5%) were never hungry. Approximately 73 percent had walkable environment while 97.4 percent had access to modern healthcare. Sixty-five percent were satisfied with quality of healthcare services, while 45.6 percent had good healthcare. Approximately 47 percent had never attended public meetings, 82.5 percent were never lonely, and 40.8 percent felt completely safe. Roughly 65 percent were satisfied with their living conditions, 52.8 percent had good overall quality of life whilst 72.5 percent were happy.

Table 2 shows the chi-square results of happiness in life and the background characteristics of the older adults. Overall, majority of the research participants (94.1%) were happy in life. Most of older adults who reported being happy were females (94.5%). Concerning age, old old (95.7%) were happier than younger old (93.5%). All older adults who were never married and separated/divorced/cohabiting were rather happier than those who were married. Older adults who were employed were happier (96.1%). Also, those who resided in urban area were much happier than those in rural setting (96.3% and 92.2%) respectively. Older adults who felt safer in the community reported being happy in life. Older adults who had higher education were much happier. Those with 2–3 and more than 6 servings of fruits and vegetables respectively, were happier. Older adults who were satisfied with their health and living conditions had better social integration, social engagement, and also had very good overall quality of life.

### Model Testing

The goodness of fit of the fitted SEM model was examined using Chi-squared test, Non-normed Fit Index (NFI), Tucker-Lewis Index (TLI), Comparative Fit Index (CFI), and Root Mean Square Error of Approximation (RMSEA). The Chi-squared statistics for the fitted models was 1916.266, (degrees of freedom = 180), with a corresponding p-value < 0.001. The NFI, TLI, CFI and RMSEA for the fitted model were 0.92, 0.95, 0.93, and 0.07, respectively. The NFI, TLI, and CFI for the fitted model were all greater than 0.90 criterion, while the RMSEA was lower than 0.08. Inferring from Byrne (2009), the goodness of fit statistics indicate that the model fits the data well.

### The Social Determinants and Happiness among Older Adults

As shown in Fig. 2, most of the indicators of social determinants of health loaded well on their latent variables. Economic stability was caused by income (*Factor Score* = 0.35, *P* < 0.01), and employment (*Factor Score* = 0.99, *P* < 0.001). Walkability *Factor Score* = 0.73, *P* < 0.001), safety (*Factor Score* = 0.49, *P* < 0.001), and place of residence (*Factor Score* = 0.81, *P* < 0.001) were significantly related to neighbourhood and built environment. Access to quality education was highly measured by higher educational attainment (*Factor Score* = 0.97, *P* < 0.001), and years educated (*Factor Score* = 0.81, *P* < 0.001). Being hungry affected access to healthy food negatively (*Factor Score*=−0.49, *P* < 0.001). However, fruit consumption (*Factor Score* = 0.73, *P* < 0.001) and vegetable consumption (*Factor Score* = 0.70, *P* < 0.001) significantly caused access to healthy food. Community engagement (*Factor Score* = 0.48, *P* < 0.05), and social integration (*Factor Score* = 0.65, *P* < 0.001) positively affected social and community context, but stress negatively caused social and community context (*Factor Score*=−0.53, *P* < 0.001).

Satisfaction with health care (*Factor Score* = 0.20, *P* < 0.001), availability of health care provider (*Factor Score* = 0.28, *P* < 0.001) and healthcare coverage (*Factor Score* = 0.39, *P* < 0.001) were positively related to health care system. Happiness of older adults was strongly caused by level of happiness (*Factor Score* = 78, *P* < 0.001) and satisfaction with living condition (*Factor Score* = 0.88, *P* < 0.001).

The social determinants of health as were analysed in this study were directly and positively related to happiness of older adults. The results show that economic stability (*β* = 0.07), neighbourhood and built environment (*β* = 0.02, *P* < 0.001), access to quality education (*β* = 0.56, *P* < 0.01), access to healthy food (*β* = 0.48, *P* < 0.001) social and community context (*β* = 0.41, *P* < 0.05), and access to quality healthcare (*β* = 0.80, *P* < 0.001) had direct relationship with happiness of older adults.

Further, the findings show that economic stability, social and community context covary (*β* = 0.45, *P* < 0.001) to affect happiness of older adults. Also, the results showed an indirect effect of healthcare system on happiness of older adults through access to healthy food (*β* = 0.54, *P* < 0.001). Also, healthy food mediated the effect of quality education on happiness of older adults (*β* = 0.84, *P* < 0.001) while access to quality education operated through neighbourhood and built environment (*β* = 0.92, *P* < 0.001) to influence happiness of older adults.

## Discussion

Enquiry into how SDOH (e.g. economic stability, access to quality education, health, neigbourhood, built environment as well as the social and community context of individuals) affect happiness of older adults has not received the requisite attention in Ghana. This study examined the relationships between the SDOH and happiness of older persons in Ghana. Direct and positive relationships are shown between the SDOH and happiness of older people. Economic stability (income and employment) of older persons was directly related to happiness, indicating the possibility of higher happiness of older persons, especially the youngest-old who are still capable of working and earning to afford their basic needs such as paying utility bills, and leisure activities. Our analysis supports results of Frey and Stutzer [9]. Diener and Biswas-Diener [47]. Blanchflower and Oswald [11] who found positive correlation between income and happiness. In some instance, income has been identified to increase happiness [12]. Similarly, evidence shows a positive relationship between employment and happiness [13]. Indicating that economic stability has positive and significant relationship with the level of happiness of older persons.

We found that neighbourhood and built environment had direct and a positive effect on happiness of older adults. Although the socio-gerontological study by Lykken [48] argues that the ability to be happy in old age is determined by intrinsic but not external factors, findings of our study possibly suggests that the external factors such as the SDOH affect happiness of older adults. Thus, the older adults who lived in better place of residence, either in the rural or urban areas and have walkable space in the neighbourhood could experience improved social relationships with neighbours, feel safer to move around freely in their communities and for leisure. Corroboratively, the experience of living in a poor neighbourhood has negative effect on happiness [25]. However, good relations with friends in the neighbourhoods positively enhance the happiness of older adults [23]. This is because the neighbourhood community represents the broader social environment that influence the level of happiness of the individual [36,37]. The environmental factors that are directly connected to happiness include access to open, natural and green spaces, which are design features that allow for social interactions [24]. Therefore, it is not just the genetics that determine happiness of older adults but also, the neighbourhood and build environment play a direct and significant role in the level of happiness among older persons.

One of the important factors that influence happiness is education [49]. Our results showed direct relationship between access to quality education and happiness of older adults, possibly indicating that older adults with higher educational attainment have higher self-confidence as well as the opportunity of getting a good job with higher income and savings which can positively make them feel happier than their colleagues who missed the educational and job opportunities. Consistent evidence shows that education has positive relationship with happiness [49–51]. Also, older persons who are highly educated, as compared to their counterparts with lower educational attainment, have higher self-confidence, decent jobs, and higher income which impact their level of happiness [27–30]. Brighouse and Swift [26] have shown that educational attainment increases level of happiness through independence and freedom of choice.

We found positive relationship between eating healthy food and happiness. This possibly explains the fact that older adults who have access to nutritious food with adequate (i.e., 3 to 5) servings of fruits and vegetables are happier than those who do not. The result of this study further confirms results of Blanchflower et al. [11] and Fararouei et al. [52] study that showed a linear relationship between happiness and the number of servings of fruits and vegetables consumed per day. Evidence shows that food that is enriched with fruits and vegetables is good for healthy living and therefore contribute to a feeling of happiness. Reasonably, low nutritional wellness has many health implications for older adults which also impact their happiness [53]. This suggestive that people who do not have access to healthy food rich in fruits and vegetables may have compromised health and happiness. Consistently, results from earlier studies show that healthy eating, specifically food rich in fruit and vegetables reduces the chances of contracting food related diseases and enhances happiness of individuals [54–56]. Similarly, evidence show a positive relationship between food and happiness [57], indicating that eating a healthy food adds to one’s happiness. Thus, there is a positive relationship between healthy eating and happiness indicating that nutritional behaviour influences happiness and for that matter, older adults [58].

Consistent with earlier studies [36, 37], our study revealed that social and community context had a direct and positive effect on happiness of older adults. Our observation is further supported by van der Have et al [31] who reported that the social and community context characteristics that are directly related to happiness include access to open social spaces for meetings and these include community centers, drinking spots for older adults, church places and game spots, which allow for social interaction among older adults. Plausible explanation could be that participation in social activities at the community level improves the sense of belonging, social contacts as well as social interaction and good relations in the neighbourhood and contribute to happiness of older adults. This corroborates with results of Davidson and Cotter [32] which showed that sense of community correlates significantly with happiness. Similarly, good relations in neighbourhoods have been identified to positively affect happiness of the individual [23]. Also, Helliwell and Putnam [33] showed that happiness is significantly related to spending time with friends and neighbours, civic participation, and trust in neighbourhoods and the local police. Meanwhile, older people are happier when they are socially engaged in the community where they live [38].

Besides, we realized that access to quality healthcare is positively related to happiness of older adults. Happiness is an important factor that contributes significantly to the efficiency of the healthcare system [15]. A plausible reason for this could be that access to quality healthcare and satisfaction with health care provision as well as the availability of healthcare provider enhance happiness of older adults. Further, Venkatapuram [18] also revealed a strong relationship between satisfaction with health and happiness. Therefore, the older adults have access to quality healthcare, the higher their happiness. Venkatapuram [18] and Howell [19] concluded that happiness can be beneficial to health and longevity. Also, good healthcare system has been recognised as a prerequisite to achieve happiness [16, 17].

The study also revealed that food mediates the effects of healthcare access and quality on happiness. This possibly explains that access to quality healthcare in a country helps to educate older people on health benefits of eating nutritious food rich in fruits and vegetables which could lead to living a happy life. Also, findings of this study show that food operate through access to quality education to impact happiness. A plausible explanation could be that higher educational attainment ensures having a good job, higher income and better understanding of nutrition with the means to afford nutritious food and therefore, improved happy life of older adults. Lastly, neighbourhood and build environment mediated the effect of quality education on happiness. This could be because higher educational attainment is influenced by social integration and engagement. Our observation is further supported the finding of a study by Ariana [59] which showed a relationship between educational attainment and social integration. However, Ariana’s finding could not establish the mediating effect between quality education and happiness of older adults through neighbourhood and build environment.

### Strengths and weaknesses

This study draws its conclusions from a nationally representative sample of aged people in Ghana. It also utilised multistage cluster sampling method in the selection of the respondents, whilst rigorous statistical analysis was conducted. The questionnaire and methods of data collection have also been validated. Moreover, the study included one more component (healthy food) to the indicators of SDOH by Healthy People and uncovers the direct and indirect effects of the social determinants of health on happiness of aged people that are important for policies and social interventions for older adults using SEM techniques. However, due to the cross-sectional nature of the survey, the study could not establish causality. Further, only variables with complete cases for this analysis were used in this study, which has the potential of producing some biased estimates. Results are generalisable to only older adults in Ghana. Lastly, the model should be tested for applicability in other countries.

## Conclusions

In examining the social determinants of happiness of older adults, this study shows that the conditions in which older adults were born, live, learn, work, play, worship, and age (SDOH) positively impact their happiness in later life. Specifically, economic stability and social and community context have significant linkages or intercorrelate to influence happiness of older adults. Most importantly, effects of quality education and healthcare on happiness of older adults was mediated by access to healthy food. Indicating that eating healthy food plays a vital role for older adults who had access to quality education and healthcare. Lastly, neighbourhood and physical environment play important role between quality education and happiness of older adults in Ghana. Whatever, social policies and interventions targeting happiness among older adults should consider the social determinants of health, most importantly, the mediating effects of food on happiness through access quality education, and healthcare system, as well as the important role that neighbourhood and build environment could play between quality education and happiness of older adults.

## Figures and Tables

**Table 1:** Background Characteristics of Respondents

Characteristic	Frequency (n)	Percentage (%)
Sex		
Male	346	44.3
Female	435	55.7
Age		
50–64	494	63.3
65–74	179	22.9
75–84	92	11.8
85 years or older	16	2.0
Marital status		
Never married	11	1.4
Married	466	59.7
Cohabiting/Separated/divorced/widowed	3	0.4
Widowed	301	38.5
Educational attainment		
Primary/JHS	336	43.0
Secondary/SHS	395	50.6
Tertiary	50	6.4
Servings of fruits		
No servings	72	9.2
< 2 servings	155	19.8
2–3 servings	390	49.9
4–5 servings	146	18.7
> 6 servings	18	2.3
Servings of vegetables		
No servings	41	5.2
< 2 servings	154	19.7
2–3 servings	440	56.3
4–5 servings	95	12.2
> 6 servings	51	6.5
Childhood residence		
Yes	600	76.8
No	181	23.2
Employment		
Yes	558	71.4
No	223	28.6
Income		
Yes	531	68.0
No	250	32.0
Hunger		
Almost every month	3	0.4
Some months but not every month	23	2.9
Only in one or two months	40	5.1
Never	715	91.5
Walkability		
Yes	567	72.6
No	214	27.4
Healthcare provider		
None	4	0.5
Modern care	761	97.4
Traditional healer	14	1.8
Home Health care	2	0.3
Quality of healthcare		
Very satisfied	124	15.9
Satisfied	511	65.4
Neutral	92	11.8
Dissatisfied	38	4.9
Healthcare coverage		
Very good	116	14.9
Good	356	45.6
Moderate	139	17.8
Bad	145	18.6
Social integration		
Never	363	46.5
1/2x per yr	263	33.7
1/2x per mo	96	12.3
1/2x per wk	41	5.2
Daily	18	2.3
Stress		
Never	644	82.5
Rarely	92	11.8
Sometimes	42	5.4
Often	3	0.4
Safety		
Completely safe	319	40.8
Very safe	277	35.5
Moderately safe	129	16.5
Slightly safe	45	5.8
Not safe	11	1.4
Satisfaction with living conditions		
Very satisfied	140	17.9
Satisfied	504	64.5
Neither	90	11.5
Dissatisfied	40	5.1
Very Dissatisfied	7	0.9
Overall quality of life		
Very good	20	2.6
Good	412	52.8
Moderate	305	39.1
Bad	35	4.5
Very bad	9	1.2
Level of happiness		
Very happy	51	6.5
Happy	566	72.5
Neither	118	15.1
Unhappy	35	4.5
Very unhappy	11	1.4

**Table 2:** Happiness by Demographics and Social Determinants of Health

Variables	Happy in life		
	% [95% CI]	(n)	P-value
Overall	94.1 [0.79,1.09]	(781)	
Sex			0.520
Male	93.6 [0.87,1.00]	(346)	
Female	94.5 [0.86,1.03]	(435)	
Age			0.814
50–64	93.5 [0.84,1.03]	(494)	
65–74	95.0 [0.91,0.99]	(179)	
75–84	95.7 [0.78,1.14]	(92)	
85+	93.8 [0.91,0.97]	(16)	
Marital status			0.827
Never married	100.0 [0.97,1.01]	(11)	
Married	94.0 [0.85,1.03]	(466)	
Separated/divorced/cohabiting	100.0 [0.93,1.05]	(3)	
Widowed	94.0 [0.88,1.00]	(301)	
Economic stability			
Income			0.813
High	94.0 [0.84,1.04]	(531)	
Low	94.4 [0.90,0.99]	(250)	
Employment			0.000
Yes	96.1 [0.85,1.07]	(558)	
No	89.2 [0.85,0.93]	(223)	
Neighbourhood and physical environment			
Housing			
Urban	96.3 [0.89,1.03]	(356)	0.015
Rural	92.2 [0.84,1.01]	(425)	
Walkability			0.141
Yes	94.5 [0.83,1.06]	(567)	
No	93.0 [0.89,0.97]	(214)	
Safety			0.016
Completely safe	91.5 [0.84,0.99]	(363)	
Very safe	96.6 [0.90,1.03]	(351)	
Moderately safe	96.1 [0.86,1.06]	(51)	
Slightly safe	100.0 [0.97,1.01]	(12)	
Not safe	75.0 [0.67,0.83]	(4)	
Educational attainment			
No formal education	81 [0.75,0.87]	(3)	0.000
Primary/JHS	90.5 [0.84,0.97]	(335)	
Secondary/SHS	97.0 [0.89,1.05]	(394)	
Tertiary	96.0 [0.86,1.06]	(49)	
Fruit consumption			
No serving	98.6 [0.84,1.13]	(72)	
< 2	93.5 [0.90,0.97]	(155)	
2–3	94.6 [0.87,1.02]	(390)	
4–5	91.8 [0.89,0.95]	(145)	
> 6	88.9 [0.85,0.92]	(18)	
Vegetable consumption			.0266
No serving	97.6 [0.90,1.06]	(41)	
< 2	90.3 [0.87,0.93]	(154)	
2–3	93.9 [0.85,1.03]	(440)	
4–5	97.9 [0.79,1.17]	(95)	
> 6	98.0 [0.88,1.08]	(51)	
Hunger			0.000
Almost every month	33.3 [0.27,0.39]	(3)	
Some month but not every month	87.0 [0.82,0.92]	(23)	
Only in a month or two months	87.5 [0.80,0.95]	(40)	
Never	95.0 [0.81,1.09]	(715)	
Health satisfaction			0.010
Very satisfied	95.2 [0.93,0.98]	(124)	
Satisfied	95.1 [0.85,1.05]	(511)	
Neutral	91.3 [0.73,1.09]	(92)	
Dissatisfied	92.1 [0.85,1.00]	(38)	
Very dissatisfied	75.0 [0.72,0.78]	(16)	
Social integration			0.014
Never	91.0 [0.84,0.98]	(363)	
Rarely	94.7 [0.90,1.00]	(263)	
Sometimes	99.0 [0.80,1.18]	(96)	
Often	100.0 [0.96,1.02]	(41)	
Very often	100.0 [0.95,1.03]	(18)	
Social engagement			0.000
Never	77.3 [0.69,0.86]	(44)	
Rarely	85.7 [0.77,0.94]	(42)	
Sometimes	97.0 [0.94,1.00]	(133)	
Often	95.1 [0.86,1.04]	(469)	
Very often	96.8 [0.79,1.15]	(93)	
Satisfaction with living condition			0.001
Very satisfied	95.7 [0.93,0.98]	(140)	
Satisfied	95.8 [0.86,1.06]	(504)	
Neutral	86.7 [0.69,1.04]	(90)	
Dissatisfied	85.0 [0.77,0.93]	(40)	
Very dissatisfied	85.7 [0.72,0.99]	(7)	
Overall quality of life			0.000
Very good	100.0 [0.95,1.03]	(20)	
Good	98.1 [0.90,1.06]	(412)	
Moderate	93.4 [0.87,0.99]	(305)	
Bad	68.6 [0.62,0.75]	(35)	
Very bad	22.2 [0.10,0.34]	(9)	
Healthcare provider availability			0.062
None	100.0 [0.91,1.07]	(4)	
Modern care	94.2 [0.79,1.09]	(761)	
Traditional healer	92.9 [0.90,0.96]	(14)	
Home health care	50 [0.46,0.54]	(2)	

## Data Availability

The dataset can be accessed by request at https://apps.who.int/healthinfo/systems/surveydata/index.php.

## Abbreviations

NPE: Neighbourhood and Built Environment
OLT: Objective List Theory
SDOH: Social determinants of health
SEM: Structural Equation Modeling
